# Nail plate split of the fifth toenail

**DOI:** 10.1016/j.jdcr.2023.06.046

**Published:** 2023-07-15

**Authors:** Kasey Cox, Julie E. Mervak

**Affiliations:** a University of Michigan Medical School, Ann Arbor, Michigan; b Department of Dermatology, University of Michigan, Ann Arbor, Michigan

**Keywords:** accessory nail, double fifth toenail, double little toenail, ectopic nail, matrixectomy, rudimentary accessory nail

## Case presentation

**Fig 1:**
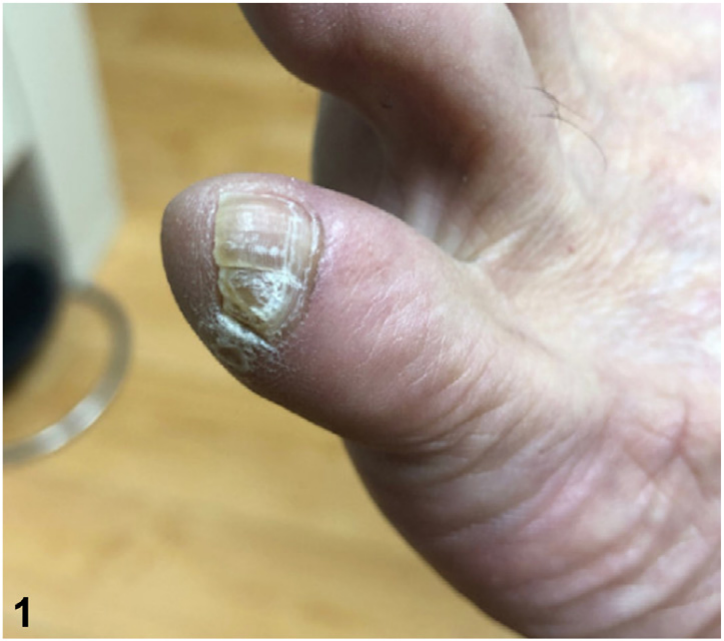

A 59-year-old man with a history of verruca vulgaris on his hands presented for evaluation of left fifth toe pain. On examination, there was a wide fifth toenail with longitudinal nail plate split. This was bilateral, but more prominent and symptomatic on the left, with the split occurring approximately one-third of the distance from the lateral edge of the nail plate (Fig 1). He noted that this had been unchanged for decades. He denied previous significant trauma or surgery to his left fifth toenail but had discomfort with shoes. To his knowledge, there was no family history of similar findings.

**Question 1: What is the most likely diagnosis?**

A. Double (accessory) fifth toenail
B. Subungual exostosis
C. Periungual fibroma
D. Subungual verruca
E. Onychophosis

**Answers:**

**Fig 2:**
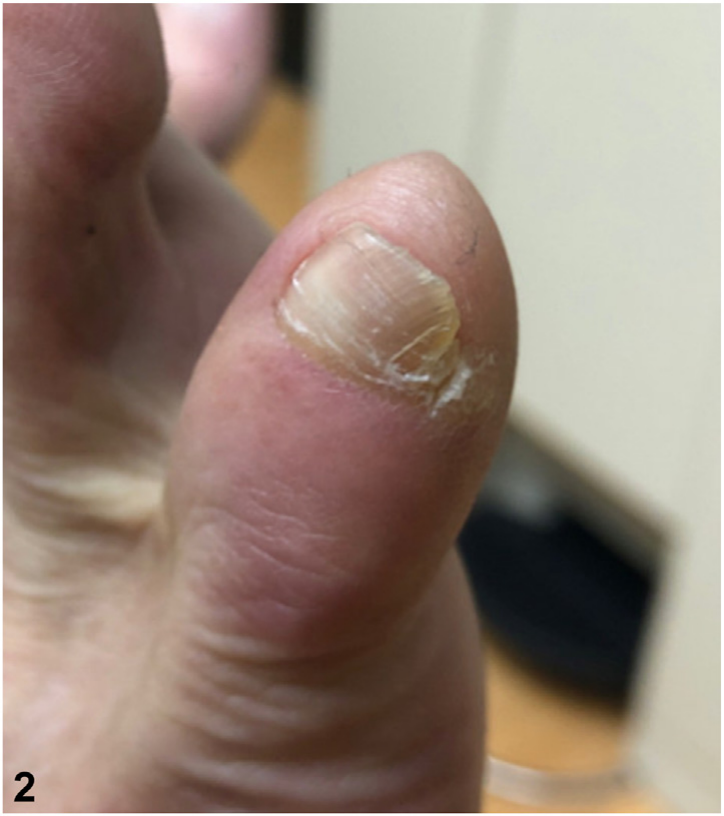
B. Subungual exostosis – Incorrect. Subungual exostosis is a benign bony tumor arising from the distal phalanx and is more common in toenails than fingernails. This presents as a nodule of the distal nail bed with accompanying onycholysis,^4^ not a nail plate split. Radiographs are confirmatory.
C. Periungual fibroma – Incorrect. Also known as Koenen’s tumors in the setting of tuberous sclerosis, this is a benign papule with hyperkeratotic tip commonly presenting at the proximal nail fold.^4^ This leads to longitudinal depression in the nail plate, and not a split nail.
D. Subungual verruca – Incorrect. Despite his history of warts, the fifth toenails did not demonstrate clinical or dermoscopic findings to support a viral etiology.
E. Onychophosis – Incorrect. Onychophosis refers to localized or diffuse hyperkeratosis at the lateral or proximal nail folds, in the space between the nail fold and nail plate, as a result of repeated minor trauma.^1^ This does not describe the nail plate abnormality.

A. Double (accessory) fifth toenail – Correct. This is considered an incomplete form of polydactyly.^1^ On examination, the nail has 2 parts, the medial being wider, with a longitudinal furrow or split. In the largest review to date of this condition, 2 completely distinct nail plates were rare, and findings were often symmetrical.^2^ A quarter of cases in this series were more prominent on one side,^2^ like our patient, with more subtle findings on the other foot (Fig 2). This is often asymptomatic but can be tender due to compression of the lateral nail fold.^3^

**Question 2: What is the next best step in diagnosis?**

A. Nail bed biopsy
B. Nail clipping for hematoxylin and eosin
C. Referral to genetics
D. Radiograph
E. Magnetic resonance imaging

**Answers:**

A. Nail bed biopsy – Incorrect. Well-developed accessory toenails, as seen in this patient, have no histologic abnormality of the nail matrix or bed. Excision shows all characteristics of a normal fifth toenail: proximal nail fold, matrix, and short nail bed.^2^
B. Nail clipping for hematoxylin and eosin – Incorrect. Similar to answer choice A, there is no histologic abnormality of well-developed accessory toenails.
C. Referral to genetics – Incorrect. Double fifth toenail is thought to be autosomal dominant, but expression is variable. There have been reports that this is common in Han Chinese,^3^ but additional reports have shown a broad range of races and ethnicities with this presentation.^2^ Double fifth toenail diagnosis alone does not require a genetics evaluation.
D. Radiograph – Correct. In a previous series, 10 patients with clinical findings of a double fifth toenail underwent foot radiographs, with 30% demonstrating a Y-shaped distal phalanx.^2^ Other reports have not shown any abnormalities on foot radiographs, but 2 nail plates and 2 matrices were seen on ultrasound.^5^ The pathophysiology of the accessory nail is not well understood. There is question of whether the presence of bone in some patients induces nail formation or if the presence of nail epithelium causes underlying mesenchymal cells to generate bone.^2^ Our patient underwent radiographs of his feet which did not show any bone abnormalities. Radiographs prior to intervention also help evaluate for other possible diagnoses such as subungual exostosis.
E. Magnetic resonance imaging – Incorrect. Advanced imaging is not necessary for diagnosis or treatment planning.

**Question 3: Should definitive treatment be desired, what is the recommended treatment approach?**

A. En-bloc excision of nail unit
B. Cotton cast placement under free edge of the nail plate
C. Urea cream twice daily
D. Avulsion and matrixectomy
E. Repeat cryotherapy

**Answers:**

**Fig 3:**
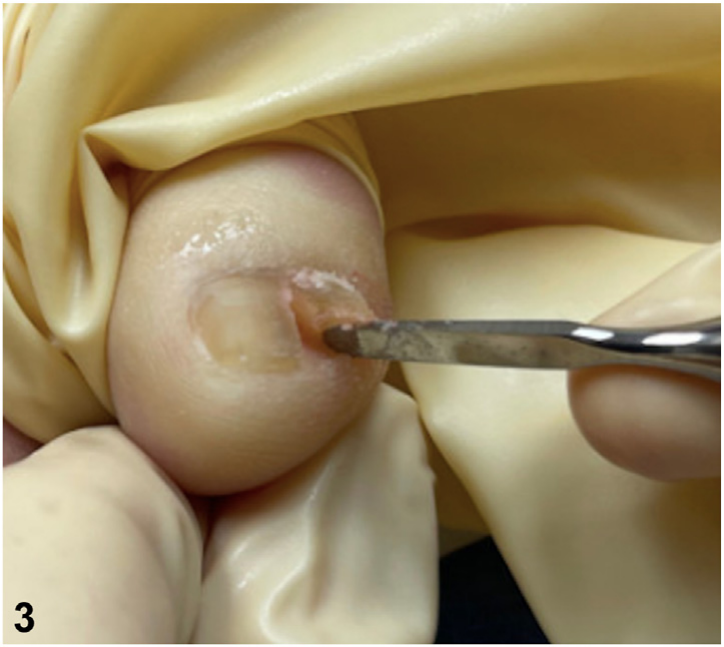
E. Repeat cryotherapy – Incorrect. Despite the patient’s history of warts, this was not the case at these sites and cryotherapy is unlikely to be curative.

A. En-bloc excision of nail unit – Incorrect. This technique is utilized for nail unit malignancies (eg, treatment of minimally invasive nail unit melanoma). As this is a benign finding, en-bloc excision is not necessary and the medial nail plate and matrix can remain intact.
B. Cotton cast placement under free edge of the nail plate – Incorrect. Although this technique can be helpful for onychocryptosis, it would not treat the underlying anatomic etiology.
C. Urea cream twice daily – Incorrect. This is a common conservative approach for nail dystrophy, including secondary to friction on the lateral aspect of the fifth toenail. This was discussed with this patient, however he elected for more definitive treatment.
D. Avulsion and matrixectomy – Correct. Although this finding is benign, it can be painful. If patients wish to pursue treatment, avulsion of the accessory toenail and application of 88% phenol to the matrix can be performed.^2^ As these are 2 independent nail plates, the avulsion is simple and only requires use of nail plate elevator and hemostat without need for nail splitter (Fig 3). This was performed for our patient with relief of his symptoms. Alternatively, surgical matrixectomy^3^ or complete surgical resection of the accessory matrix, proximal nail fold, and hyponychium can be performed,^2^ particularly if there is any doubt about the diagnosis and a pathology specimen is desired.

## Conflicts of interest

None disclosed.
